# Supplementary material for: Resolving the full spectrum of human genome variation using Linked-Reads
Source: Genome Res. 2019 Apr;29(4):635–45. doi: 10.1101/gr.234443.118 (PMC6442396; doi:10.1101/gr.234443.118)
Supplement: Supplemental Material [file supp_gr.234443.118_Supplemental_longranger-2.2.zip › longranger-2.2.tar.bz2!/lr2.2-public/lib/stan/cmdstan/stan/doc/api/html/agrad_2fwd_2matrix_2rows__dot__product_8hpp_source.html]

Stan: src/stan/agrad/fwd/matrix/rows\_dot\_product.hpp Source File


|  |  |
| --- | --- |
|  | Stan  2.5.0  probability, sampling & optimization |


- Main Page
- Related Pages
- Namespaces
- Classes
- Files

- File List
- File Members

All Classes Namespaces Files Functions Variables Typedefs Enumerations Enumerator Friends Macros Pages

- src
- stan
- agrad
- fwd
- matrix

rows\_dot\_product.hpp

Go to the documentation of this file.

1 #ifndef STAN\_\_AGRAD\_\_FWD\_\_MATRIX\_\_ROWS\_\_DOT\_PRODUCT\_HPP

2 #define STAN\_\_AGRAD\_\_FWD\_\_MATRIX\_\_ROWS\_\_DOT\_PRODUCT\_HPP

3

4 #include <vector>

5 #include <stan/math/matrix/Eigen.hpp>

6 #include <stan/math/matrix/typedefs.hpp>

7 #include <stan/math/error\_handling/matrix/check\_matching\_dims.hpp>

8 #include <stan/agrad/fwd/matrix/typedefs.hpp>

9 #include <stan/agrad/fwd/matrix/dot\_product.hpp>

10 #include <stan/agrad/fwd/fvar.hpp>

11

12

13 namespace stan {

14  namespace agrad {

15

16  template<typename T, int R1,int C1,int R2, int C2>

17  inline

18  Eigen::Matrix<fvar<T>, R1, 1>

19  rows\_dot\_product(const Eigen::Matrix<fvar<T>, R1, C1>& v1,

20  const Eigen::Matrix<fvar<T>, R2, C2>& v2) {

21  stan::math::check\_matching\_dims("rows\_dot\_product(%1%)",v1,"v1",

22  v2,"v2",(double\*)0);

23  Eigen::Matrix<fvar<T>, R1, 1> ret(v1.rows(),1);

24  for (size\_type j = 0; j < v1.rows(); ++j) {

25  Eigen::Matrix<fvar<T>,R1,C1> crow1 = v1.row(j);

26  Eigen::Matrix<fvar<T>,R2,C2> crow2 = v2.row(j);

27  ret(j,0) = dot\_product(crow1, crow2);

28  }

29  return ret;

30  }

31

32  template<typename T, int R1,int C1,int R2, int C2>

33  inline

34  Eigen::Matrix<fvar<T>, R1, 1>

35  rows\_dot\_product(const Eigen::Matrix<double, R1, C1>& v1,

36  const Eigen::Matrix<fvar<T>, R2, C2>& v2) {

37  stan::math::check\_matching\_dims("rows\_dot\_product(%1%)",v1,"v1",

38  v2,"v2",(double\*)0);

39  Eigen::Matrix<fvar<T>, R1, 1> ret(v1.rows(),1);

40  for (size\_type j = 0; j < v1.rows(); ++j) {

41  Eigen::Matrix<double,R1,C1> crow = v1.row(j);

42  Eigen::Matrix<fvar<T>,R2,C2> crow2 = v2.row(j);

43  ret(j,0) = dot\_product(crow, crow2);

44  }

45  return ret;

46  }

47

48  template<typename T, int R1,int C1,int R2, int C2>

49  inline

50  Eigen::Matrix<fvar<T>, R1, 1>

51  rows\_dot\_product(const Eigen::Matrix<fvar<T>, R1, C1>& v1,

52  const Eigen::Matrix<double, R2, C2>& v2) {

53  stan::math::check\_matching\_dims("rows\_dot\_product(%1%)",v1,"v1",

54  v2,"v2",(double\*)0);

55  Eigen::Matrix<fvar<T>, R1, 1> ret(v1.rows(),1);

56  for (size\_type j = 0; j < v1.rows(); ++j) {

57  Eigen::Matrix<fvar<T>,R1,C1> crow1 = v1.row(j);

58  Eigen::Matrix<double,R2,C2> crow = v2.row(j);

59  ret(j,0) = dot\_product(crow1, crow);

60  }

61  return ret;

62  }

63  }

64 }

65 #endif

check\_matching\_dims.hpp

typedefs.hpp

stan::agrad::fvar

**Definition:** fvar.hpp:13

typedefs.hpp

stan::math::check\_matching\_dims

bool check\_matching\_dims(const char \*function, const Eigen::Matrix< T1, R1, C1 > &y1, const char \*name1, const Eigen::Matrix< T2, R2, C2 > &y2, const char \*name2, T\_result \*result)

**Definition:** check\_matching\_dims.hpp:15

dot\_product.hpp

stan::agrad::size\_type

Eigen::Matrix< double, Eigen::Dynamic, Eigen::Dynamic >::Index size\_type

**Definition:** typedefs.hpp:14

stan::agrad::dot\_product

fvar< T > dot\_product(const Eigen::Matrix< fvar< T >, R1, C1 > &v1, const Eigen::Matrix< fvar< T >, R2, C2 > &v2)

**Definition:** dot\_product.hpp:20

fvar.hpp

Eigen.hpp

stan::agrad::rows\_dot\_product

Eigen::Matrix< fvar< T >, R1, 1 > rows\_dot\_product(const Eigen::Matrix< fvar< T >, R1, C1 > &v1, const Eigen::Matrix< fvar< T >, R2, C2 > &v2)

**Definition:** rows\_dot\_product.hpp:19


---

[ Stan Home Page ]

*© 2011–2014,
Stan Development Team.*
